# Stent Ductal Oscilante na Atresia Pulmonar Valvar

**DOI:** 10.36660/abc.20190829

**Published:** 2020-11-01

**Authors:** Arun Gopalakrishnan, Kavassery Mahadevan Krishnamoorthy, Paidi Suresh Kumar, Sivasankaran Sivasubramonian

**Affiliations:** 1 Sree Chitra Tirunal Institute for Medical Sciences and Technology – Cardiology ThiruvananthapuramKerala Índia Sree Chitra Tirunal Institute for Medical Sciences and Technology – Cardiology, Thiruvananthapuram, Kerala – Índia

**Keywords:** Cardiopatias Congênitasas, Atresia Pulmonar, Anomalia de Ebstein, Permeabilidade do Canal Arterial, Ecocardiografia/métodos

Um recém-nascido a termo de 2,7 kg foi diagnosticado no terceiro dia de vida com anomalia de Ebstein da válvula tricúspide, regurgitação tricúspide grave, atresia pulmonar valvar e circulação pulmonar ducto-dependente. O índice cardiotorácico era de 90% e o escore GOSE de 1,48. A saturação de oxigênio era de 65% e melhorou para 88% com 0,005 mcg/kg/min de prostaglandina. A pressão sistólica do ventrículo direito era de 40mmHg, com uma pressão arterial sistólica de 65mmHg. A criança foi encaminhada para implante de stent no ducto arterial no 7° dia de vida, após confirmação de atresia pulmonar anatômica. O ducto arterial longo recebeu stents de 3,5x16mm e 3,5x8mm, utilizando um acesso 4F na artéria femoral direita. Ambas as extremidades pulmonar e aórtica estavam bem cobertas e a saturação sistêmica melhorou para 85% com ar ambiente e sem prostaglandina.

**Figura 1:**
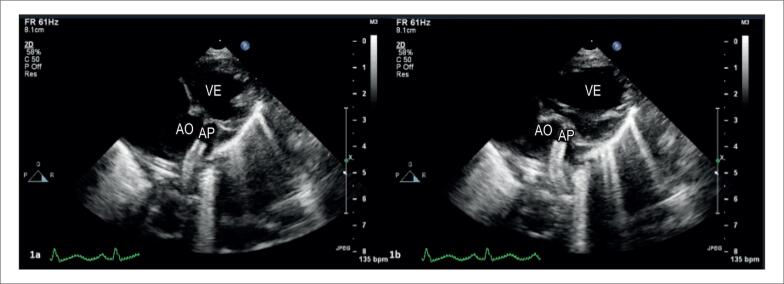
Imagens estáticas da ecocardiografia transtorácica do paciente em projeção paraesternal de eixo curto modificada na sístole (painel 1a) e diástole (painel 1b) mostram diferença marcante no eixo longo do stent no ducto arterial.

A ecocardiografia pós-procedimento confirmou stent bem posicionado e com bom fluxo. No entanto, o stent parecia oscilar a cada ciclo cardíaco (figura 1, vídeo 1). Este “sinal de oscilação do stent” foi confirmado na angiografia aórtica pós-implante de stent (vídeo 2). A atresia pulmonar valvar está associada ao “sinal da gaivota” bem descrito na angiografia. O acúmulo de contraste na artéria pulmonar principal que retém uma ligação fibrosa ao tecido anular pulmonar leva ao sinal da gaivota, com os ramos das artérias pulmonares assumindo o formato de asas de “gaivota”. A atresia pulmonar valvar geralmente está associada a um ducto arterial reto inserido no tronco da artéria pulmonar em metade dos casos.^1^ Mesmo quando o ducto é longo e tortuoso nos outros, estudos sugerem que ele é mais facilmente manejado durante o implante de stent ductal na atresia pulmonar valvar do que na atresia pulmonar de segmento longo.^2^^,^^3^ A oscilação do stent ductal na atresia pulmonar valvar a cada ciclo cardíaco sugere que a extremidade pulmonar do stent está dentro da artéria pulmonar principal, que é uma estrutura intrapericárdica e reflete fielmente as contrações mecânicas cardíacas.^4^ Sugerimos que este achado ecocardiográfico pode ser utilizado para avaliação da posição do stent em relação à extremidade pulmonar na atresia pulmonar valvar.

**Vídeo 1:**
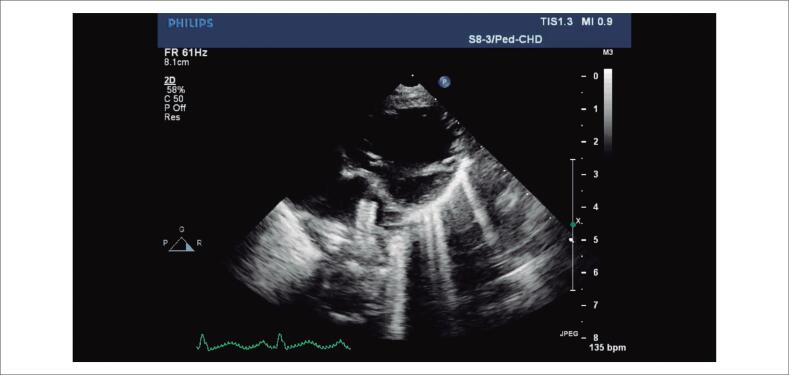
– A ecocardiografia transtorácica na projeção paraesternal de eixo curto modificada mostra o stent oscilante no ducto arterial. A extremidade aórtica do stent é fixa, enquanto a movimentação vigorosa do segmento da artéria pulmonar principal na atresia pulmonar valvar é responsável pela oscilação. link: http://abccardiol.org/supplementary-material/2020/11505/2019-0829-video-1.mp4

**Vídeo 2:**
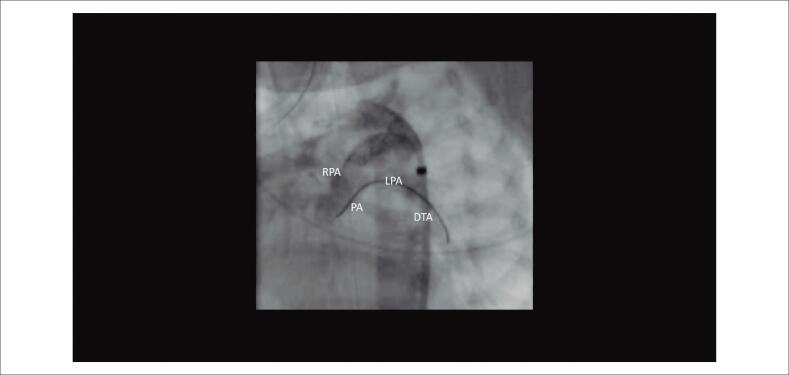
– Fluoroscopia em loop na projeção oblíqua anterior esquerda mostrando o ducto arterial com stent e o “sinal da gaivota” e “sinal do stent oscilante” da atresia pulmonar valvar. link: http://abccardiol.org/supplementary-material/2020/11505/2019-0829-video-2.mp4

O risco potencial de movimento cíclico do stent foi avaliado e o bebê foi mantido em acompanhamento rigoroso. Ele está bem aos 5 meses de acompanhamento, com bom fluxo de stent, artérias pulmonares de bom tamanho e os movimentos oscilatórios do stent persistem.
