# Supplementary material for: Stent Ductal Oscilante na Atresia Pulmonar Valvar
Source: Arq Bras Cardiol. 2020 Nov 1;115(5):961–3. [Article in Portuguese] doi: 10.36660/abc.20190829 (PMC8452223; doi:10.36660/abc.20190829)
Supplement: Translation

# Translation: Oscillating Ductal Stent in Valvar Pulmonary Atresia

**Authors:** Arun Gopalakrishnan, Kavassery Mahadevan Krishnamoorthy, Paidi Suresh Kumar, Sivasankaran Sivasubramonian

**Affiliations:** 1 ThiruvananthapuramKerala Índia Sree Chitra Tirunal Institute for Medical Sciences and Technology – Cardiology, Thiruvananthapuram, Kerala – Índia

**Keywords:** Heart Defects, Congenital, Pulmonary Atresia, Ebstein Anomaly, Ductus Arteriosus, Patent, Echocardiography/methods

A 2.7 kg term neonate was diagnosed on day 3 of life with Ebstein's anomaly of the tricuspid valve, severe tricuspid regurgitation, valvar pulmonary atresia and duct-dependent pulmonary circulation. The cardiothoracic ratio was 90%, and GOSE score was 1.48. Oxygen saturation was 65% and improved to 88% on 0.005 mcg/kg/min of prostaglandin. The right ventricular systolic pressure was 40mmHg against a systolic blood pressure of 65mmHg. The child was taken up for stenting of the arterial duct on day 7 of life after the confirmation of anatomic pulmonary atresia. The long arterial duct was stented with 3.5x16mm and 3.5x8mm stents, using a 4F right femoral artery access. Both the pulmonary and aortic ends were well covered and systemic saturation improved to 85% on room air and off prostaglandin.

The post-procedure echocardiography confirmed a well-positioned stent with good flow. However, the stent appeared to oscillate with every cardiac cycle (figure 1, video 1). This “oscillating stent sign” was confirmed on the post-stenting aortic angiogram (video 2). Valvar pulmonary atresia is associated with the well-described “seagull sign” on angiogram. Pooling of contrast in the main pulmonary artery that retains a fibrous attachment to pulmonary annular tissue leads to the seagull sign, with the branch pulmonary arteries assuming the shape of the wings of the ‘seagull’. Valvar pulmonary atresia is generally associated with a straight arterial duct inserted into the main pulmonary artery in half of the cases.^1^ Even when the duct is long and tortuous in the others, studies suggest that it is more readily negotiated during ductal stenting in valvar pulmonary atresia as opposed to long segment pulmonary atresia.^2^^,^^3^ The oscillation of the ductal stent in valvar pulmonary atresia with every cardiac cycle suggests that the pulmonary end of the stent is within the main pulmonary artery, which is an intrapericardial structure and faithfully reflects the mechanical contractions of the heart.^4^ We suggest that this echocardiographic finding may be utilized for the assessment of stent position with respect to the pulmonary end in valvar pulmonary atresia.

**Figure 1:** Transthoracic echocardiographic still images of the patient in modified parasternal short axis projection in systole (panel 1a) and diastole (panel 1b) shows marked difference in the long axis of the stent in the arterial duct.

**Video 1:** – Transthoracic echocardiography in the modified parasternal short axis projection shows the oscillating stent in the arterial duct. The aortic end of the stent is fixed, while the vigorous movement of the main pulmonary artery segment in valvar pulmonary atresia is responsible for the oscillation. link: http://abccardiol.org/supplementary-material/2020/11505/2019-0829-video-1.mp4

**Vídeo 2:** – Transthoracic echocardiography in the modified parasternal short axis projection shows the oscillating stent in the arterial duct. The aortic end of the stent is fixed, while the vigorous movement of the main pulmonary artery segment in valvar pulmonary atresia is responsible for the oscillation. link: http://abccardiol.org/supplementary-material/2020/11505/2019-0829-video-2.mp4

The potential risk of cyclic stent movement was assessed, and the baby was kept on close follow up. He is doing well at 5 months’ follow up with good stent flow, good sized pulmonary arteries and the oscillatory movements of the stent persist.
